# A Rare Case of Breast Implant-Associated Diffuse Large B-Cell Lymphoma

**DOI:** 10.1155/2019/1801942

**Published:** 2019-11-27

**Authors:** Christopher Larrimore, Annmarie Jaghab

**Affiliations:** Nova Southeastern University Dr. Kiran C. Patel College of Osteopathic Medicine, USA

## Abstract

This is a case of an elderly female who presented for follow-up ultrasound of the right breast after routine mammogram revealed a small benign mass. A subsequent ultrasound detected a small nodular mass that was described as benign in appearance. Although the patient was asymptomatic, a fine-needle biopsy was performed to rule out malignancy. Results from immunohistochemistry and FISH studies of the biopsy were positive for diffuse large B-cell lymphoma (DLBCL). The patient underwent surgery for lumpectomy and removal of breast implants. Intraoperative tissue samples were analyzed by pathology using both flow cytometry and microscopy, and results confirmed DLBCL. With total tumor resection and implant removal completed, the patient did not require additional treatments as the prognosis of DLBCL status post implant removal is excellent. She returned for follow-up six months later and has since had no signs of reoccurrence.

## 1. Introduction

Primary breast lymphoma has a low rate of occurrence comprising an estimated 0.5% of breast malignancies [1, 2]. Even rarer are lymphomas associated with breast implants with the majority of the documented cases being T-cell lymphomas. Worldwide, over 500 cases of anaplastic large cell lymphoma associated with breast implants have been documented [3], including 4 cases of cutaneous T-cell lymphoma [1]. However, to date, only 5 cases of diffuse large B-cell lymphoma (DLBCL) associated with breast implants have been reported [1, 2, 4]. Because lymphomas associated with breast implants have been reported as well localized, implant removal and mass resection have proven to be satisfactory treatment. However, without many cases documented within the literature and without long-term outcome studies, it is difficult to know if additional treatments are required. In this case report, a patient with a newly diagnosed DLBCL is described. In the report, her clinical presentation, diagnostic studies, and outcome after surgery will be discussed.

## 2. Case Report

A 70-year-old female without significant past medical history presented to the clinic after the results of a routine mammogram revealed the continued presence of a small mass in her right breast. A mammogram dated 3 years prior detected this mass and reported it as small and benign, located inferior to an implant of her right breast. The more recent mammogram findings reported no change in size. The patient was asymptomatic and denied previous history of cancer and no family history of breast cancer, or other malignancies. She denied weight loss, fever, night sweats, or change in appetite. However, she did report mild discomfort with self-palpation to her lower right breast. Her breast implants were placed 28 years ago in Argentina for cosmetic reasons and have not since been revised. Additionally, she reported a long history of smoking tobacco daily.

**Figure 1:**
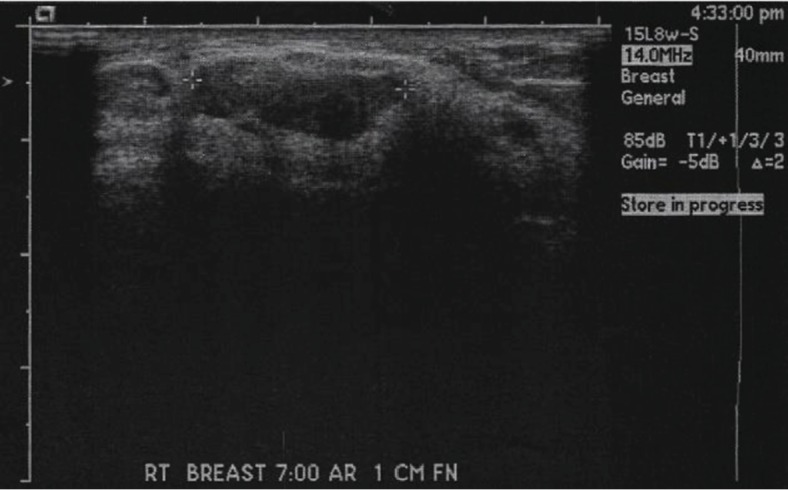
Ultrasound of the right breast before fine-needle biopsy shows a nodular density with multiple areas of hyperechoic densities within it.

To rule out malignancy, an ultrasound was performed and confirmed the presence of an elongated nodular density benign in appearance. The mass had multiple areas of hyperechoic densities within it and was located at the 8 o'clock position 10 cm from the right nipple. The size was measured to be 3.4 × 1.3 × 3.2 cm, with no evidence of shadowing or implant rupture reported (Figure 1). The impression of the ultrasound was the presence of a hamartoma, a benign lesion of the breast that corresponded with previous mammogram results. Despite recommendations for a follow-up mammogram at a later date, the patient was referred by her primary care provider for fine-needle biopsy and samples were sent for evaluation.

Biopsy samples were sent to pathology for analysis using histology, immunohistochemistry, and FISH studies. Histologic sections of the core biopsy fragments showed malignant lymphoma of diffuse pattern. The tumor cells were large in size with anaplastic and focally spindled morphology. Additionally, there were increased mitotic figures and cellular apoptosis. Immunohistochemistry revealed lymphoma cells positive for CD20, PAX5, BCL2, BCL6, and vimentin and negative for CD3, CD5, CD10, cyclin D1, smooth muscle myosin, S100, CD31, CD20, E-cadherin, and keratin. It was determined that the neoplasm had a nongerminal center phenotype with a proliferative index of 80-90%. FISH studies were completed to determine the presence of MYC, BCL2, or BCL6 gene rearrangements. All were negative; however, an abnormal signal pattern suggestive of gains of BCL2 was detected. Overall, testing of the fine-needle biopsy confirmed DLBCL.

The patient underwent surgery for lumpectomy of the right breast, as well as bilateral breast implant removal. During the operation, the presence of a calcified capsule surrounding each nontextured implant was noted. Found within the capsules was silicone. It was unclear if the nontextured implants had ruptured prior to surgery or if there was a rupture during the procedure. Both capsules were removed, and silicone was irrigated and aspirated from the sites. The tumor was found to be in direct contact with the calcified capsule but without evidence of capsule invasion into the tumor. The tumor was also widely excised with ample samples sent to pathology for further testing. The patient was given a one-time dose of cefazolin 1 gm IV and hydrocodone for pain management. She was discharged 2 days later with a complaint of moderate breast pain that was being managed well with hydrocodone.

Intraoperative tissue samples were sent to pathology. The breast lesion was noted to be well circumscribed and consisted of monomorphous cells with large lymphocytic features. No evidence of invasion from the calcified capsule was found. The cells had pleomorphic ovoid to round nuclei with occasional atypical mitotic figures. Atypical cells, as well as foamy macrophages, were present in the touch prep. Atypical lymphoid cells with anaplastic nuclear features were noted to infiltrate into collagenized-sclerotic stroma and at times adjacent fatty lobules. Immunohistochemistry was positive for CD45, BCL2, BCL6, CD43, CD79A, MUM1, and PAX5. Flow cytometry results, in addition to the histologic and immunohistochemistry staining, confirmed the diagnosis of DLBCL. The tumor was ultimately measured as 2.3 × 1.3 cm in maximal dimension, and the benign sclerotic capsule tissue provided evidence of the chronic rupture of implants.

The patient returned to the clinic 2 weeks after surgery and had well-healing scars without signs of infection. She returned again 6 months later and reported no pain at the surgical sites. Physical exam did not find any lymphadenopathy or mass with palpation. She continues to remain in good health.

## 3. Discussion

In this report, a rare case of DLBCL associated with breast implants is presented. The mass was first detected 3 years prior during routine mammogram. Findings reported the mass to be benign in appearance, which resulted in no additional work-up. Three years later, routine mammogram reported no change in size or appearance. While the patient continued to be asymptomatic, it was decided to order additional testing to rule out any potential malignancy. Fine-needle biopsy with ultrasound guidance was performed, and pathology results revealed the diagnosis of DLBCL.

DLBCL is a non-Hodgkin lymphoma (NHL). It is the most common type of lymphoid malignancy in adults and is B-cell in origin [1, 2]. In western countries, DLBCL accounts for 31% of NHLs [2]. While DLBCL does have a higher rate of occurrence in adults, its association with breast implants is extremely rare. With only 0.5% of breast malignancy being comprised of primary breast lymphomas [1], only 5 cases of DLBCL have been reported [1, 2, 4, 7]. The more frequent primary breast lymphoma is anaplastic large cell lymphoma (ALCL). This lymphoma is T-cell in origin, is associated most commonly with textured implants, and has been reported in over 500 cases worldwide [2, 3]. Regardless, both forms of primary breast lymphomas are rarely documented in the medical literature resulting in the pathogenesis being poorly understood.

NHLs are characterized by the abnormal growth of lymphocytes. In ALCL, the expression of T-cell markers CD4 and CD43 occurs in 82% and 77% of the cases, respectively [2]. Also present is a typical expression of CD30 and a characteristic horseshoe-shaped nucleus [2, 3]. While ALCL can be further divided into two groups based upon the presence of anaplastic lymphoma kinase (ALK), implant-associated ALCL is primarily ALK-negative [2, 3]. In contrast, DLBCL is heterogeneous in respect to morphology and biomarker expression. Based upon gene expression studies, DLBCL has been subdivided into separate molecular subtypes that arise at different stages of B-cell differentiation [5]. Although there is diversity in marker expression as outlined in Table 1, the more common diagnostic markers for DLBCL include BCL2, BCL6, c-MYC, and PAX5 [2, 5, 6]. With the detection of these cellular markers and the presence of specific morphologic changes, ALCL and DLBCL can be properly identified.

In this patient, the microscopy of the tissue samples from the fine-needle biopsy showed malignant lymphoma in a diffuse pattern. The tumor cells present were large in size and with an anaplastic morphology. DLBCL morphology can be diverse with variants that include centroblastic, immunoblastic, T-cell/histiocyte rich, or anaplastic [2]. To confirm the diagnosis, testing that included flow cytometry and immunohistochemistry of both the fine-needle biopsy and the intraoperative tissue samples was completed. Laboratory testing was consistently positive for CD45, BCL2, BCL6, CD43, CD79A, MUM1, and PAX5. All of these are markers that strongly correlate with DLBCL [2, 5, 7]. Further reinforcing the diagnosis was the lack of T-cell markers that would be present in ALCL. This included CD3 and CD5.

While DLBCL is an aggressive cancer and rapidly fatal if left untreated, DLBCL associated with breast implants has a far better prognosis and is often less aggressive. Nonbreast implant-associated DLBCL requires a treatment combination of cyclophosphamide, doxorubicin, vincristine, and prednisone (CHOP). Adding rituximab, a chimeric monoclonal antibody against CD20, has proven to increase survival rates [8]. Breast implant-associated DLBCL, if localized, requires no CHOP regimen. Instead, treatment is the complete resection of malignant tissue and removal of breast implants. If malignancy has invaded the lymph nodes, a treatment regimen should be considered.

In this case, a 70-year-old female with 28-year-old nontextured silicone breast implants developed a slow-growing mass in the right breast later identified as DLBCL. While symptomatology is dependent upon the severity of the disease, the common clinical presentation for DLBCL is breast swelling and tenderness. This patient was asymptomatic with benign mammogram findings. Because tumors associated with breast implants can be aggressive and are sometime associated with squamous cell tumors, additional testing was ordered that ultimately revealed the malignancy. Within 6 months from diagnosis, the patient underwent complete tumor resection and implant removal. There were no reported intraoperative or postoperative complications. Subsequent follow-up appointments at 2 weeks, 6 months, and 1 year were highly suggestive of a successful treatment as no new mass was noted and the patient remained asymptomatic.

## Figures and Tables

**Table 1:** A listing of DLBCL diagnostic markers [4, 6].

Biomarker expression/genetic alterations	Percentage of cases
CD19, CD20, CD22, CD79A, CD45	Pan B-cell antigens, highly expressed
CD30	25% (anaplastic variant)
BCL2	25-80%
BCL6	70%
CD10	30-60%
MUM1/IRF4	35-65%
CD5	Uncommon, aggressive disease
(14;18) translocation	30%
MYC gene rearranged	5-15%
